# Bayesian Analysis of a Quantile Multilevel Item Response Theory Model

**DOI:** 10.3389/fpsyg.2020.607731

**Published:** 2021-01-08

**Authors:** Hongyue Zhu, Wei Gao, Xue Zhang

**Affiliations:** ^1^School of Mathematics and Statistics, Northeast Normal University, Changchun, China; ^2^China Institute of Rural Education Development, Northeast Normal University, Changchun, China

**Keywords:** multilevel item response theory, quantile regression, Bayesian analysis, Gibbs sampling, non-normality of latent variable

## Abstract

Multilevel item response theory (MLIRT) models are used widely in educational and psychological research. This type of modeling has two or more levels, including an item response theory model as the measurement part and a linear-regression model as the structural part, the aim being to investigate the relation between explanatory variables and latent variables. However, the linear-regression structural model focuses on the relation between explanatory variables and latent variables, which is only from the perspective of the average tendency. When we need to explore the relationship between variables at various locations along the response distribution, quantile regression is more appropriate. To this end, a quantile-regression-type structural model named as the quantile MLIRT (Q-MLIRT) model is introduced under the MLIRT framework. The parameters of the proposed model are estimated using the Gibbs sampling algorithm, and comparison with the original (i.e., linear-regression-type) MLIRT model is conducted via a simulation study. The results show that the parameters of the Q-MLIRT model could be recovered well under different quantiles. Finally, a subset of data from PISA 2018 is analyzed to illustrate the application of the proposed model.

## Introduction

Multilevel or hierarchical linear models are used widely in educational and psychological researches (e.g., Raudenbush, 1988; Goldstein, 1995; Snijders and Bosker, 1999). These models allow data to be collected at different levels; for example, test data are obtained from students, students are nested within schools, and so on. Item response theory (IRT) models can be plugged in multilevel models, known as multilevel IRT (MLIRT) models. In educational research and educational assessment, the MLIRT model is widely applied, where the main objective is to investigate the relationship between covariates (e.g., student’s social background, school financial resources) and outcome variables (e.g., student’s ability; Fox, 2010).

Adams et al. (1997) noted that a two-level IRT model could be seen as a multilevel perspective on item response modeling. In this formulation, we can divide the two-level IRT model in two components: the measurement part with the respective IRT measurement model and the structural part with the respective regression model. Furthermore, Kamata (2001) and Fox and Glas (2001) extended the two-level IRT model to three levels, with both studies proposing a two-level regression model (i.e., student-level and group-level covariates were analyzed in different levels) on the ability parameters as the structural model. Because the explanatory variables might not always be measured accurately, Fox and Glas (2003) showed how to model latent explanatory variables with measurement errors within the MLIRT model. Additionally, MLIRT models can also be applied to handle more-complicated factors such as longitudinal response data and response times. For example, Huang (2015); Schmidt et al. (2016) introduced MLIRT models for longitudinal data to assess the changes in students’ abilities over time. Also, when jointly modeling the responses and response times, the ability and speed parameters can be considered as outcome variables of a multivariate multilevel model for various analyses (e.g., Entink et al., 2009; Fox, 2010).

Regarding the estimation of MLIRT models, studies have shown that all the model parameters can be estimated simultaneously using a fully Bayesian method, regardless of whether the IRT model is a two-parameter model for dichotomous response data or a graded response model for polytomous response data (Fox, 2005; Natesan et al., 2010). Compared with the traditional two-stage estimation procedure, this estimation procedure in the MLIRT models leads to a proper treatment of the measurement error associated with the ability parameter (Adams et al., 1997; Fox and Glas, 2001, 2003; Fox, 2004, 2005, 2010). See Fox (2010) for more advantages of the MLIRT modeling framework.

One fact that needs to be noted is that the distribution of latent variables in the IRT model is often assumed to be normal (e.g., Bock and Lieberman, 1970; Bock and Aitkin, 1981; Wirth and Edwards, 2007). This assumption may be reasonably when students follow a normally distributed population, but in many cases, it is not satisfied (e.g., Woods, 2006, 2015; Woods and Thissen, 2006; Sass et al., 2008). It has been shown that when a non-normal latent distribution is assumed to be normal, estimates of IRT model parameters are biased (e.g., Stone, 1992; Sass et al., 2008; Woods, 2015). This issue has also been explored regarding multidimensional and graded response models (e.g., Finch, 2010, 2011; Svetina et al., 2017; Wang et al., 2018). However, there has been little attention to the non-normality of latent variables in the MLIRT framework.

On the other hand, the structural models in the MLIRT models are always linear regression (LR) approaches. Herein, LR generally assumes that the errors are normally distributed and should be homoscedastic, which are not often in accordance with practice, because of non-normality of latent variables or heterogeneity of errors (Harvey, 1976; Koenker and Bassett, 1978, 1982; Jarque and Bera, 1980; Tsionas, 2003). It is also well known that a LR model mainly focuses on the relation between explanatory variables and the conditional mean of latent variables, however, it is quite possible that the relationship between variables may vary at different points along the response distribution, researchers also might be interested in the relationships between variables on various locations of the distribution, such as on one or both of the tails of the distribution.

These problems can be addressed by the quantile regression (QR) (Koenker and Bassett, 1978), which is a valuable and robust tool for analyzing the conditional quantile functions of latent variables. When considering the relationship between explanatory variables and latent variables, QR allows to compute several different regression curves corresponding to the various percentage points of the distribution and thus obtain a more complete relationship between them. In addition, QR is regarded as a robust method in regression because the estimation results are insensitive to outliers or the non-normality of response distributions. Overview of QR methods, one can refer to Yu et al. (2003); Koenker and Ng (2005). As such, QR has been used by many researchers in the field of education (e.g., Wößmann, 2005; Haile and Nguyen, 2008; Chen and Chalhoub-Deville, 2014; Giambona and Porcu, 2015; Gürsakal et al., 2016; Costanzo and Desimoni, 2017). The aim of most studies has been to scrutinize inequality of opportunity in education through investigating the determinants of student achievement, or to analyze individual and/or family background determinants of student achievement with respect to test results.

In recent years, QR has also been applied successfully in latent variable models (e.g., Dunson et al., 2003; Allen and Powell, 2011; Burgette and Reiter, 2012; Wang et al., 2016; Feng et al., 2017; Belloni et al., 2019; Liu et al., 2020). Burgette and Reiter (2012) considered QR in a factor analysis model to analyze the effects of latent variables on the lower quantiles of the response distribution. Wang et al. (2016) introduced QR into a structural equation model (SEM) to assess the conditional quantile of the outcome latent variable given the explanatory latent variables and covariates. Liu et al. (2020) proposed a quantile hidden Markov model to examine the entire conditional distribution of the response given the hidden state and potential covariates. Factor analysis models can be converted to IRT models under possible parameterizations (Kamata and Bauer, 2008; Wang and Zhang, 2019). Therefore, it is reasonable and implementable to apply QR to MLIRT models.

This study is motivated by the MLIRT models focus largely on the relationship between variables at the mean level, which may result in neglecting potential differences across the response distribution. Herein, taking a two-level (i.e., item level and student level) IRT model as a demonstration, we embed the QR approach in a MLIRT model to obtain what we refer to as the quantile MLIRT (Q-MLIRT) model. This approach enables us to investigate the relationship between explanatory variables and latent variables at various locations of the response distribution, as well as having minimal assumptions on error terms, which is a flexible and applicable approach in general. Moreover, the Q-MLIRT model can be estimated in a Bayesian framework with a Markov chain Monte Carlo (MCMC) algorithm. It should be emphasized that the Q-MLIRT model is not a replacement but a supplement to the conventional MLIRT model. If the relationship between explanatory variables and latent variables on the entire distribution is of interest, Q-MLIRT is a good recommendation.

The rest of this paper is organized as follows. The proposed Q-MLIRT model is introduced in Section “Model Description,” the MCMC algorithm is presented in Section “Bayesian Estimation,” and a simulation study conducted to evaluate the empirical performance of the proposed method is reported in Section “Simulation Study.” A real data study to demonstrate the use of Q-MLIRT models is reported in Section “Empirical Example,” and finally a discussion and suggestions for further research are presented in Section “Discussion.”

## Model Description

Before describing the Q-MLIRT model considered here, we revisit MLIRT models briefly. We consider only a two-level IRT model, namely an item level and a student level. As presented by Adams et al. (1997), the two-level IRT model consists of two components: the measurement part—an IRT measurement model—which describes the probability of observed responses conditional upon the latent variable, and the structural part—a LR model—which describes the between-student variation in the latent variable. The LR model is on ability parameters, where the ability parameter is dependent on the covariates:

(1) $$\theta_{i}={\text{X}}_{i}^{T}\,\beta +\delta_{i},$$

where θ_*i*_ is the latent trait (i.e., ability) of student i (*i* = 1,…,*n*), **X**_i_ is a **q** × 1 vector of known covariates (e.g., gender, socioeconomic status, and major) for student **i**, and β is the corresponding q × 1 vector of regression coefficients. We assume that δ_*i*_ are independently and identically normally distributed with mean zero and variance α^2^. In this form, a student’s achievement or performance is represented as ability parameter θ_*i*_, and the relationship between students’ abilities and the involved explanatory variables is reflected in the regression coefficients β. Note that the coefficients of all covariates are treated as fixed effects here.

LR analysis measures the relationship between explanatory variables and students’ abilities by modeling a conditional mean function of θ_*i*_. Given explanatory variables **X**_i_, then:

(2) $$E\,\left(\theta_{i}|{\text{X}}_{i}\right)={\text{X}}_{i}^{T}\,\beta .$$

That is, the conditional distribution of θ_*i*_ is assumed to be a linear combination of covariates **X**_*i*_ with normally distributed errors. In usual LR approaches, the data are used to find out a single regression line that minimizes the sum of squared errors (least squared estimation) to estimate the relationship. Consequently, the focus is on average performance of response variables about covariates. However, as mentioned above, the errors may be non-normally distributed or even heteroscedastic in reality, and when a distribution is asymmetric (e.g., heavy-tailed or skewed), the mean is not the center of the distribution, while the median is likely to be a more appropriate measure of central tendency than the mean (Edgeworth, 1888; Fox, 1997; Koenker and Ng, 2005; Hao and Naiman, 2007; Trafimow et al., 2018). What is more, the relation between explanatory variables and latent variables may be different across the entire distribution. As a result, the conditional mean fails to meet the research needs in many cases. Therefore, we must provide an elaborate or specific description of the inter-relationship among latent variables and explanatory variables.

A more appropriate analysis can be achieved from conditional quantiles of θ_*i*_. QR provides a suitable method for modeling conditional quantile functions (Koenker and Hallock, 2001). Under a number of different quantiles τ ∈ (0,1), we say the τth quantile of δ_*i*_ is the value *q*_τ_, for which *p*(δ_*i*_ < *q*_τ_) = τ, that is *Q*_τ_(δ_*i*_) = *q*_τ_. The τth conditional quantile of θ_*i*_ can be then expressed as:

(3) Qτ⁢(θi|Xi)=XiT⁢βτ,

where β_τ_ is a **q** × 1 vector of QR coefficients depend on τ, and it reflects the relationship between **X**_i_ and the τth conditional quantile of θ_*i*_, which means the marginal effects of the explanatory variables may differ over quantiles of the distribution of θ_*i*_. Since numerous quantiles (e.g., 25%, 50%, 75%) are modeled in QR, it is possible to understand the relationship between variables at various locations of the response distribution. For example, when τ = 0.5, XiT⁢βτ is the conditional median of θ_*i*_, and β_τ_ reflects the relationship between explanatory variables and the conditional median of θ_*i*_. In QR approaches, the classical inference of β_τ_ is through a minimization of the weighted sum of absolute residuals for all the observations.

Therefore, θ_*i*_ can be assessed in the following QR-type structural model, by the τth conditional quantile of θ_*i*_ plus an error term:

(4) $$\theta_{i}={\text{X}}_{i}^{T}\,\beta_{\tau }+\delta_{i}.$$

Unlike in the LR model [i.e., Eq. (1)], here the distribution of δ_*i*_ is not specified. The only assumption is that the τth quantile of δ_*i*_ is zero, that is *q*_τ_ = 0, so that we have Eq. (3). In other words, the QR structural model does not rely on any parametric specification of the conditional distribution of θ_*i*_. Note that when the error term δ_*i*_ in Eq. (4) is normally or symmetrically distributed, results of LR and QR at the median (τ = 0.5) are consistent, in other words, the MLIRT model of Eq. (1) is a special version of the Q-MLIRT model with normality of δ_*i*_ and τ = 0.5.

Therefore, the two-level Q-MLIRT model can be defined by combining Eq. (4) with an IRT model, namely:

(5) $$P\left(Y_{i\,k}=1|\theta_{i},a_{k},b_{k}\right)=\Phi \left(a_{k}\left(\theta_{i}-b_{k}\right)\right),\theta_{i}={\text{X}}_{i}^{T}\,\beta_{\tau }+\delta_{i},$$

where we adopt a two-parameter normal ogive (2PNO) model in the first level. In this article, only dichotomously scored items are considered. Here, Φ denotes the cumulative standard normal distribution function, Y_ik_ = 1 means a correct response of student *i* on item *k*(*k* = 1,…*K*), and *a*_*k*_ and *b*_*k*_ are the discrimination and difficulty parameters, respectively, of item *k*. The second level is QR on the ability parameters with an arbitrary quantile τ ∈ (0,1), and the parameters have the same meanings as those in Eq. (4).

Herein, we fix the item parameters of one item as known (i.e., *a_1=1* and *b_1=0*) to identify the Q-MLIRT model as did Fox and Glas (2001). In the subsequent analysis, we show that the proposed model offers a more complete view of the relationship among explanatory variables and latent variables at various locations of the response distribution, including the central tendency, upper tail, and lower tail. In many cases, the error terms are non-normally distributed or heterogeneous, which fails to meet the assumption of LR, but nevertheless QR is also suitable for such cases.

## Bayesian Estimation

We adopt the Bayesian estimation approach in the QR structural model (e.g., Yu and Moyeed, 2001; Lee and Neocleous, 2010; Reich et al., 2011; Wang et al., 2016). Combined with the Gibbs sampling method for 2PNO models (Albert, 1992), a fully Bayesian approach is presented to estimate the parameters of the Q-MLIRT model. Similar to Wang et al. (2016), we adopt the asymmetric Laplace distribution1 (ALD^1^; Yu and Moyeed, 2001) to approximate the distribution of error terms in the QR structural model. More details about the ALD and the Bayesian QR method are presented in Appendix. Specifically, we assume δ_*i*_∼*ALD*(0,ω,τ), then θ_*i*_ follows $A\,L\,D\,\left({\text{X}}_{i}^{T}\,\beta_{\tau },\omega ,\tau \right)$. As shown by Reed and Yu (2009); Kozumi and Kobayashi (2011), if a random variable follows *ALD* (μ,ω,τ) then it can be represented as a normal distribution, so we obtain:

(6) $$\theta_{i}|\beta_{\tau },\omega ,e_{i}\,\overset{i\,n\,d}{∼}N\,\left({\text{X}}_{i}^{T}\,\beta_{\tau }+k_{1}\,e_{i},k_{2}\,\omega \,e_{i}\right),$$

where $k_{1}=\frac{1-2\,\tau }{\tau \,\left(1-\tau \right)}$, $k_{2}=\frac{2}{\tau \,\left(1-\tau \right)}$, and **e** = {*e*_1_,*e*_2_,⋯,*e*_*n*_} follows an exponential distribution with scale parameter ω. That is to say, the parameter space is augmented by a latent variable e. This mixed representation of $A\,L\,D\,\left({\text{X}}_{i}^{T}\,\beta_{\tau },\omega ,\tau \right)$ enables Bayesian inference based on the Gibbs sampling algorithm to estimate the parameters of the QR structural model.

### Gibbs Sampling

From Eq. (5), the observations consist of the item responses **Y** in the first level and the explanatory variables **X** in the second level. As a result, the full posterior distribution of the parameters given the observed data is:

(7) $$p\left(Z,\theta ,a,b,\beta_{\tau },e,\omega |Y,X\right)\propto \prod_{i=1}^{n}(\left(\prod_{k=1}^{K}p\left(z_{i\,k}|\theta_{i},a_{k},b_{k},y_{i\,k}\right)\right)p\left(\theta_{i}|\beta_{\tau },e_{i},\omega ,{\text{X}}_{i}\right))p\,\left(\text{e}|\omega \right)\,p\,\left(a\right)\,p\,\left(b\right)\,p\,\left(\beta_{\tau }\right)\,p\,\left(\omega \right),$$

where the augmented data *z*_*ik*_ follow:

$$p\left(z_{i\,k}|\theta_{i},a_{k},b_{k},y_{i\,k}\right)\propto \varphi \left(z_{i\,k};a_{k}\left(\theta_{i}-b_{k}\right),1\right)[I\left(z_{i\,k}>0\right)I\left(y_{i\,k}=1\right)+I\left(z_{i\,k}\leq 0\right)I\left(y_{i\,k}=0\right)],$$

where φ is the normal probability density function (PDF) and I(⋅) is an indicator function.

The following conjugate prior distributions of the model parameters are used:

$$a_{k}∼N\left(\mu_{a},\sigma_{a}^{2}\right)I\left(a_{k}>0\right),$$

$$b_{k}∼N\,\left(\mu_{b},\sigma_{b}^{2}\right),$$

$$\beta_{\tau }∼N_{q}\,\left(\Lambda_{0\,\beta },{\text{H}}_{0\,\beta }\right),$$

$$\omega^{-1}∼G\,a\,m\,m\,a\,\left(\alpha_{0\,\omega },\beta_{0\,\omega }\right),$$

where μ_*a*_, σ_*a*_, μ_*b*_, σ_*b*_, Λ_0β_, **H**_**0**β_, α_0ω_, and β_0ω_ are hyperparameters. The detailed procedure of the Gibbs sampling algorithm is summarized below.

Step 1: Sampling *z*_*ik*_. Let ξ be the vector of all item parameters, given the parameters θ and ξ, the variables *z*_*ik*_ are independent. For *i* = 1,…,*n* and *k* = 1,…,*K*, the fully conditional posterior distribution of z_ik_ can be written as:

(8) (zi⁢k|θ,ξ,y)∼{N⁢(ak⁢(θi-bk),1)⁢truncated⁢at⁢the⁢left⁢by⁢ 0⁢if⁢yi⁢k=1N⁢(ak⁢(θi-bk),1)⁢truncated⁢at⁢the⁢right⁢by⁢ 0⁢if⁢yi⁢k=0 .

Step 2: Sampling *a*_*k*_. According to the principle of conjugate distribution and combined with the prior of *a*, the fully conditional posterior density of *a_k* follows:

(9) $$\left(a_{k}|{\text{z}}_{k},\theta ,b_{k}\right)\;∼N\,\left(\frac{\frac{\mu_{a}}{\sigma_{a}^{2}}+\sum_{i=1}^{n}\text{[}z_{i\,k}\left(\theta_{i}-b_{k}\right)]}{\frac{1}{\sigma_{a}^{2}}+\sum_{i=1}^{n}{\left(\theta_{i}-b_{k}\right)}^{2}},\frac{1}{\frac{1}{\sigma_{a}^{2}}+\sum_{i=1}^{n}{\left(\theta_{i}-b_{k}\right)}^{2}}\right).$$

Step 3: Sampling *b_k*. The fully conditional posterior density of *b*_*k*_ is:

(10) (bk|zk,θ,ak)∼N⁢(μbσb2-∑i=1n(zi⁢k-ak⁢θi)⁢ak1σb2+n⁢ak2,11σb2+n⁢ak2).

Step 4: Sampling θ_*i*_. The ability parameters are independent given other parameters. Using Eqs. (6) and (8), it follows that:

(11) $$f\left(\theta_{i}|{\text{z}}_{i},\xi ,\beta_{\tau },\omega ,e_{i}\right)\propto f\left({\text{z}}_{i}|\theta_{i},\xi \right)f\left(\theta_{i}|\beta_{\tau },\omega ,e_{i}\right),$$

Eq. (11) is a normal model, where θ_*i*_ has a normal prior parameterized by β_τ_,*e*_*i*_, and ω. So the fully conditional posterior density of θ_*i*_ is given by:

(12) (θi|zi,ξ,βτ,ω,ei)∼N⁢(μθσθ2+∑k=1K(zi⁢k+ak⁢bk)⁢ak1σθ2+∑k=1Kak2,11σθ2+∑k=1Kak2),

where $\mu_{\theta }={\text{X}}_{i}^{T}\,\beta_{\tau }+k_{1}\,e_{i}$, $\sigma_{\theta }^{2}=k_{2}\,\omega \,e_{i}$.

Step 5: Sampling ω. Letting *v* = ω^−1^ and using the gamma conjugate prior density for *v* and the PDF of $A\,L\,D\,\left({\text{X}}_{i}^{T}\,\beta_{\tau \,\tau },\omega ,\tau \right)$, the fully conditional posterior distribution of *v* is given by:

$$p\,\left(v|\theta ,\beta_{\tau }\right)\propto v^{n+\alpha_{0\,\omega }-1}\,e\,x\,p\,\left\{-\left[\beta_{0\,\omega }+\sum_{i=1}^{n}\rho_{\tau }\,\left(\theta_{i}-{\text{X}}_{i}^{T}\,\beta_{\tau }\right)\right]\,v\right\},$$

from which we have:

(13) $$\left(\omega^{-1}|\theta ,\beta_{\tau }\right)∼G\,a\,m\,m\,a\,\left(n+\alpha_{0\,\omega },\beta_{0\,\omega }+\sum_{i=1}^{n}\rho_{\tau }\,\left(\theta_{i}-{\text{X}}_{i}^{T}\,\beta_{\tau }\right)\right).$$

Step 6: Sampling *e*_*i*_. From Eq. (6) together with an exponential distribution of *e_i* with scale parameter ω, the fully conditional posterior distribution of ei-1 follows an inverse Gaussian distribution.

(14) (ei-1|θi,βτ,ω) ∼I⁢n⁢v⁢e⁢r⁢s⁢e-G⁢a⁢u⁢s⁢s⁢i⁢a⁢n⁢(2⁢k2+k12|θi-XiT⁢βτ|,2⁢k2+k12k2⁢ω).

Step 7: Sampling β_τ_ . Because Eq. (6) is a normal distribution conditionally on *e_i*, it is straightforward to derive the fully conditional posterior distribution of β_τ_, which is given by:

(15) $$\left(\beta_{\tau }|\theta ,e,\omega \right)∼N\,\left(\mu_{\beta },\Sigma_{\beta }^{-1}\right),$$

where $\mu_{\beta }=\sum_{\beta }^{-1}\left({\text{H}}_{0\,\beta }^{-1}\,\Lambda_{0\,\beta }+\sum_{i=1}^{n}\frac{\left(\theta_{i}-k_{1}\,e_{i}\right)\,X_{i}}{k_{2}\,\omega \,e_{i}}\right)$ and $\sum_{\beta }={\text{H}}_{0\,\beta }^{-1}+\sum_{i=1}^{n}\frac{X_{i}\,X_{i}^{T}}{k_{2}\,\omega \,e_{i}}$.

## Simulation Study

In this section, a simulation study is carried out to evaluate the performance of the Q-MLIRT model. We evaluate the parameter recovery with the Gibbs sampler. The Gibbs sampling algorithm is implemented in MATLAB (MathWorks, 2016), and the source code is available to readers upon request.

### Simulation Design

Two covariates X_1_ and X_2_ are considered, which are independent standard normal variables. The latent variable θ_*i*_(*i* = 1,…,*n*) is generated from three different models:

*Case 1:* θ_*i*_ = β_1_*X*_*i*1_ + β_2_*X*_*i*2_ + δ_*i*_, δ_*i*_∼*N*(0, 0.5)

*Case 2:* θ_*i*_ = β_1_*X*_*i*1_ + β_2_*X*_*i*2_−ρ_τ_|β_1_*X*_*i*1_ + β_2_*X*_*i*2_| + |β_1_*X*_*i*1_ + β_2_*X*_*i*2_|δ_*i*_, δ_*i*_∼*N*(0, 1)

*Case 3:*θ_*i*_ = β_1_*X*_*i*1_ + β_2_*X*_*i*2_ + δ_*i*_. δ_*i*_∼*Gamma*(0.5, 1)−0.5

Parameters β_1_ and β_2_ are set to 0.5. Case 1 represents a normal and homoscedastic error model, the assumption of which conforms to that of LR models, case 2 represents a heteroscedastic error model, and case 3 represents a skewed error model. Of these, cases 2 and 3 evaluate the Q-MLIRT model with heavy-tailed and non-normal latent variables, respectively. For cases 1 and 3, the conditional τth quantile of θ takes the form *Q*_τ_(θ|**X**_1_,**X**_2_) = β_0τ_ + β_1τ_**X**_1_ + β_2τ_**X**_2_, where β_0τ_ = ρ_τ_, β_1τ_ = 0.5, β_2τ_ = 0.5 for τ ∈ (0,1), and ρ_τ_ is the τth quantile of δ_*i*_. For case 2, the conditional quantile function is *Q*_τ_(θ|**X**_1_,**X**_2_) = β_1_**X**_1_ + β_2_**X**_2_−ρ_τ_|β_1_**X**_1_ + β_2_**X**_2_| + ρ_τ_|β_1_**X**_1_ + β**X**_2_| = β_1τ_**X**_1_ + β_2τ_**X**_2_, where β_1τ_ = 0.5 and β_2τ_ = 0.5 for τ ∈ (0,1).

In order to estimate the QR coefficients β_1τ_ and β_2τ_ under different quantiles, and compare with their theoretical values 0.5, three quantile levels (i.e., τ = 0.25, 0.5, and 0.75) are chosen, which represent the lower, central, and upper tail of the response distribution, respectively. We consider three sample sizes (i.e., *n* = 500, 1000, 2000) and two test lengths (i.e., *K* = 20, 40). Overall, there are 54 conditions (3 structural models × 3 quantiles × 3 sample sizes × 2 test lengths). Response patterns are generated by the 2PNO model, with the discrimination parameters generated from *U*(0.5, 1.5) and the difficulty parameters generated from *N*(0,0.5).

For the conjugate normal prior of β_τ_, we set the prior mean **Λ**_0β_ = (0, 0, 0) and the covariance matrix **H**_**0**β_ is 100 times an identity matrix; which is a type of weakly informative prior. For the conjugate inverse gamma prior of ω, we set α_0ω_ = 28 and β_0ω_ = 4. For the prior of item parameters a and b, we set μ_*a*_ = 0, σ_*a*_ = 200 and μ_*b*_ = 0, σ_*b*_ = 100, respectively.

The Gibbs sampling estimation procedure of the Q-MLIRT model was iterated 10,000 times. A burn-in period of 5,000 iterations was used, and the parameter estimate was the mean of the posterior distribution of the parameter. The convergence of the Gibbs sampler was checked by the Gelman–Rubin method (Gelman and Rubin, 1992), and the values of the potential scale reduction factor (PSRF) in the burn-in period were less than 1.10 for all parameters. Overall, 100 replications were conducted across all simulation conditions.

A related issue must be considered in the application of Q-MLIRT, namely that the estimates of item parameters differ for different quantiles because of the fact that the fully conditional posterior distribution of item parameters depends on τ. As can be seen from Eq. (6), the location and scale of θ_*i*_ are changed in the estimation process of item parameters under different quantiles. In this case, bias may not make sense, a large bias does not mean poor estimates, and the results of different quantiles are also not comparable. We expect the estimates of item parameters under different quantiles are close to each other after some transformation. To this end, we introduce a new measurement that is actually suitable for situations in which item parameters are estimated on different IRT scales, to further illustrate the similarity and comparability between them.

It is known that if θ_*i*_ have a transformation relationship on two scales, namely:

$$\theta_{i}^{*}=p\,\theta_{i}+t,$$

where *p* and *t* are constants, then the relationships between the item parameters can be formulated as:

$$a_{k}^{*}=\frac{a_{k}}{p},$$

$$b_{k}^{*}=p\,b_{k}+t.$$

In this case, we can use similarity functions, such as the cosine function, to measure the similarity of item parameters at different scales. Two vectors are defined to be similar if the distance between them is small, which is measured by the cosine of the angle between them. Let **a** = (*a*_1_,…,*a*_*k*_) and ${\text{a}}^{*}=\left(a_{1}^{*},\ldots ,a_{k}^{*}\right)$ be the discrimination parameter vectors on two different scales, then the cosine similarity between the two vectors is defined as:

(16) $$\cos\left(a,{\text{a}}^{*}\right)=\frac{\sum_{k}a_{k}\,a_{k}^{*}}{\sqrt{\sum_{k}{\left(a_{k}\right)}^{2}}\,\sqrt{\sum_{k}{\left(a_{k}^{*}\right)}^{2}}}.$$

If $a_{k}^{*}=\frac{a_{k}}{p}$, then *cos*⁡(**a**,a^*^) = 1. Similarly, the cosine similarity between two difficulty parameter vectors **b** = (*b*_1_,…,*b*_*k*_) and ${\text{b}}^{*}=\left(b_{1}^{*},\ldots ,b_{k}^{*}\right)$ can be defined as:

(17) $$\cos\left(b,{\text{b}}^{*}\right)=\frac{\sum_{k}\left(b_{k}-E\,\text{b}\right)\,\left(b_{k}^{*}-E\,{\text{b}}^{*}\right)}{\sqrt{\sum_{k}{\left(b_{k}-E\,\text{b}\right)}^{2}}\,\sqrt{\sum_{k}{\left(b_{k}^{*}-E\,{\text{b}}^{*}\right)}^{2}}},$$

where *E***b** and *E*b* are the means of the elements in vectors **b** and **b**^∗^, respectively. If $b_{k}^{*}=p\,b_{k}+t$, then *cos*⁡(**b**,b^*^) = 1.

In this sense, if the cosine of the angle between the true-parameter vector and the estimated-parameter vector is close to 1, it can be concluded that these two vectors are very similar and close, and the estimates can also be called “good estimates;” conversely, if the cosine is away from 1, it indicates that these two vectors are very different, and the estimates can also be called “bad estimates.” The higher the cosine similarity, the closer they are. Note that the “good” and “bad” estimates are just defined in the context of the transformation relationship that we considered as above, and accuracy of all the item parameters’ estimations is measured by the cosine similarity. The upshot is, when the estimated results of item parameters under each quantile are close to each other and are both good (i.e., the calculated cosine similarity of item parameters under each quantile is close to 1), we say that the item parameters are well recovered and that any group of estimates can be selected. Otherwise, the recovery of item parameters is poor and the results of different quantiles are not comparable to one another to some extent.

Given that there is no existing work on Q-MLIRT in the literature, we compare the estimated results with the two-level structural IRT model denoted in Eq. (1), where the structural model is a LR on θ_*i*_. Hereafter, we refer to it as the mean regression multilevel IRT (M-MLIRT) model. We estimate it through a fully Bayesian estimation procedure that is easily extracted from the estimation procedure in Fox and Glas (2001). We use the average root-mean-square error (RMSE), the average bias, and the average cosine similarity to evaluate parameter recovery.

### Simulation Results

Tables 1, 2 summarize the parameter recovery of the item parameters for the Q-MLIRT model in comparison to the M-MLIRT model for small and large test lengths respectively. In general, the item parameters are estimated accurately in all the cases (i.e., cases 1, 2, and 3). For each sample size, estimates of the item parameters of the Q-MLIRT model under different quantiles are close to the true parameter values, reflected mainly in the facts that (i) the cosine similarities between the true-parameter vector and the estimated-parameter vectors under different quantiles are all very close to 1 and (ii) the RMSEs are all small. In addition, when the sample size increases from 500 to 2,000, the cosine similarities increase, the RMSE and the bias in almost cases decrease for the estimates of item parameters. Results show that with increasing test length, the accuracy of almost all item parameters’ estimations also has some improvement, this could because we can get more accurate information from students as they answer more items.

**TABLE 1:** Summary of parameter recovery of item parameters when *K* = 20.

Case	n	Method	τ	a			b		
				Cos	Bias	RMSE	Cos	Bias	RMSE
1	500	Q	0.25	0.994	0.069	0.162	0.985	0.010	0.115
			0.5	0.994	0.124	0.198	0.987	–0.004	0.112
			0.75	0.994	0.074	0.171	0.986	–0.009	0.116
		M		0.995	0.024	0.158	0.992	–0.002	0.118
	1,000	Q	0.25	0.997	0.042	0.114	0.992	0.007	0.082
			0.5	0.997	0.050	0.111	0.993	2E–04	0.077
			0.75	0.997	0.032	0.111	0.992	–0.006	0.083
		M		0.997	2E–04	0.112	0.996	0.001	0.071
	2,000	Q	0.25	0.998	0.020	0.084	0.996	0.002	0.059
			0.5	0.999	0.030	0.079	0.996	–0.009	0.056
			0.75	0.998	0.020	0.080	0.996	–0.014	0.061
		M		0.999	0.003	0.070	0.997	4E–04	0.047
2	500	Q	0.25	0.993	0.054	0.163	0.984	0.038	0.102
			0.5	0.992	0.108	0.194	0.986	–0.003	0.099
			0.75	0.993	0.056	0.166	0.982	–0.037	0.107
		M		0.992	0.031	0.197	0.985	−4E–04	0.105
	1,000	Q	0.25	0.996	0.052	0.130	0.991	0.046	0.089
			0.5	0.996	0.064	0.131	0.993	0.004	0.071
			0.75	0.996	0.051	0.123	0.991	–0.039	0.084
		M		0.997	0.021	0.107	0.992	3E–04	0.070
	2,000	Q	0.25	0.998	0.043	0.097	0.995	0.049	0.075
			0.5	0.998	0.032	0.089	0.996	0.003	0.054
			0.75	0.998	0.045	0.096	0.996	–0.041	0.071
		M		0.998	0.011	0.083	0.996	3E–04	0.053
3	500	Q	0.25	0.994	0.015	0.139	0.986	–0.003	0.111
			0.5	0.994	0.067	0.162	0.986	–0.015	0.108
			0.75	0.992	0.062	0.175	0.984	–0.025	0.116
		M		0.993	–0.003	0.144	0.987	0.021	0.106
	1,000	Q	0.25	0.997	–0.006	0.096	0.993	0.013	0.078
			0.5	0.997	0.039	0.118	0.993	–0.008	0.082
			0.75	0.995	0.018	0.124	0.991	–0.007	0.082
		M		0.996	–0.014	0.124	0.995	0.027	0.078
	2,000	Q	0.25	0.999	0.009	0.074	0.996	–0.005	0.055
			0.5	0.998	0.035	0.089	0.996	–0.016	0.061
			0.75	0.997	0.020	0.096	0.995	–0.023	0.067
		M		0.998	–0.013	0.098	0.997	0.023	0.061

*Q, Q-MLIRT model; M, M-MLIRT model.*

**TABLE 2:** Summary of parameter recovery of item parameters when *K* = 40.

Case	n	Method	τ	a			b		
				Cos	Bias	RMSE	Cos	Bias	RMSE
1	500	Q	0.25	0.995	0.051	0.147	0.986	0.019	0.111
			0.5	0.995	0.096	0.169	0.985	–0.001	0.112
			0.75	0.995	0.079	0.159	0.987	–0.026	0.111
		M		0.995	0.011	0.146	0.985	0.005	0.114
	1,000	Q	0.25	0.997	0.039	0.113	0.993	0.001	0.078
			0.5	0.998	0.059	0.121	0.992	0.003	0.082
			0.75	0.997	0.039	0.113	0.993	–0.012	0.079
		M		0.998	–0.003	0.091	0.993	0.002	0.073
	2,000	Q	0.25	0.999	0.022	0.075	0.996	0.009	0.060
			0.5	0.999	0.030	0.075	0.997	0.002	0.054
			0.75	0.999	0.022	0.074	0.996	–0.003	0.058
		M		0.999	0.009	0.070	0.997	–0.002	0.047
2	500	Q	0.25	0.993	0.054	0.167	0.984	0.029	0.106
			0.5	0.994	0.097	0.183	0.985	0.005	0.099
			0.75	0.993	0.067	0.161	0.983	–0.032	0.102
		M		0.994	–0.002	0.152	0.984	–0.002	0.099
	1,000	Q	0.25	0.997	0.038	0.115	0.991	0.037	0.082
			0.5	0.997	0.042	0.108	0.993	0.001	0.067
			0.75	0.997	0.047	0.119	0.991	–0.037	0.081
		M		0.996	–0.001	0.113	0.991	0.003	0.071
	2,000	Q	0.25	0.998	0.030	0.088	0.996	0.040	0.068
			0.5	0.998	0.030	0.084	0.996	–0.001	0.049
			0.75	0.998	0.032	0.088	0.996	–0.039	0.067
		M		0.998	–0.007	0.080	0.996	–0.002	0.046
3	500	Q	0.25	0.995	6E–05	0.139	0.986	–0.005	0.116
			0.5	0.995	0.069	0.160	0.985	–0.010	0.112
			0.75	0.994	0.064	0.158	0.985	–0.029	0.117
		M		0.995	0.019	0.148	0.985	0.025	0.099
	1,000	Q	0.25	0.997	–0.005	0.101	0.994	2E–05	0.084
			0.5	0.997	0.047	0.117	0.993	–0.008	0.081
			0.75	0.997	0.030	0.116	0.993	–0.015	0.084
		M		0.997	0.021	0.114	0.994	0.022	0.073
	2,000	Q	0.25	0.999	–0.005	0.070	0.996	1E–04	0.057
			0.5	0.999	0.015	0.071	0.996	–0.008	0.056
			0.75	0.998	0.012	0.082	0.996	–0.013	0.061
		M		0.998	5E–04	-0.082	0.996	0.027	0.057

*Q, Q-MLIRT model; M, M-MLIRT model.*

From Tables 1, 2, it is also indicated that, as for the estimation of item parameters, the item parameters’ estimations of the M-MLIRT model and the Q-MLIRT model are close in each case, mainly from the reflect of the cosine similarities and RMSEs. It should be noted that although for case 2 and case 3, the distribution of ability is non-normal, it seems not to have effects on the estimation of item parameters for the MLIRT model. Specifically, for case 3, the bias of the difficulty parameters for the M-MLIRT model are always larger than the Q-MLIRT model, but from the cosine similarity and RMSE points of view, the estimates of the difficulty parameters for the M-MLIRT model and Q-MLIRT model are close. The relationship between the cosine similarity and the bias, and how they behave in more different situations, are worthy of further study and discussion.

Tables 3, 4 give the estimated results of the structural regression coefficients for the Q-MLIRT model in comparison with those of the M-MLIRT model. First, it can be concluded that the regression coefficients are estimated accurately in all the cases (cases 1, 2, and 3) for the Q-MLIRT model. When the sample size increases from 500 to 2,000, the RMSEs decrease with increasing sample size, which means that the estimates are increasingly stable. The bias of regression coefficients also decreases obviously with increasing sample size, especially when τ = 0.5. The trends are less obvious for quantiles 0.25 and 0.75, mainly because there are few samples at the upper and lower tails (τ = 0.75 and 0.25, respectively) of the conditional distribution of θ_*i*_. Nevertheless, the directions of the bias are increasingly consistent with increasing sample size, especially when *n* = 2,000, which means that the estimates tend to true and stable values. From Table 4, the results show that as larger test length leads to more accurate estimations of the structural regression coefficients, mainly reflecting in the decreased biases and RMSEs in almost all conditions. Together with the estimation of item parameters, it can be concluded that the increase of test length will improve the accuracy of parameter estimation of the Q-MLIRT model, especially for the structural regression coefficients.

**TABLE 3:** Bayesian estimates of regression coefficients when *K* = 20.

Case	n	Method	τ	β_1τ_		β_2τ_	
				Bias	RMSE	Bias	RMSE
1	500	Q	0.25	–0.030	0.080	–0.036	0.081
			0.5	–0.049	0.084	–0.046	0.084
			0.75	–0.030	0.081	–0.032	0.083
		M		0.021	0.089	0.015	0.088
	1,000	Q	0.25	–0.029	0.060	–0.026	0.064
			0.5	–0.017	0.055	–0.025	0.056
			0.75	–0.028	0.061	–0.026	0.060
		M		0.007	0.058	0.008	0.058
	2,000	Q	0.25	–0.021	0.045	–0.018	0.046
			0.5	–0.016	0.041	–0.015	0.043
			0.75	–0.025	0.047	–0.021	0.046
		M		0.004	0.041	0.005	0.039
2	500	Q	0.25	–0.043	0.080	–0.044	0.081
			0.5	–0.068	0.095	–0.073	0.096
			0.75	–0.046	0.086	–0.046	0.089
		M		–0.054	0.096	–0.062	0.099
	1,000	Q	0.25	–0.046	0.069	–0.049	0.070
			0.5	–0.049	0.071	–0.047	0.070
			0.75	–0.047	0.068	–0.047	0.068
		M		–0.067	0.082	–0.067	0.082
	2,000	Q	0.25	–0.042	0.054	–0.046	0.058
			0.5	–0.034	0.051	–0.037	0.052
			0.75	–0.047	0.057	–0.048	0.060
		M		–0.069	0.076	–0.070	0.076
3	500	Q	0.25	0.012	0.068	0.013	0.065
			0.5	–0.016	0.068	–0.010	0.067
			0.75	–0.032	0.080	–0.030	0.079
		M		0.016	0.082	0.011	0.080
	1,000	Q	0.25	0.017	0.051	0.013	0.051
			0.5	–0.012	0.054	–0.010	0.054
			0.75	–0.018	0.057	–0.023	0.061
		M		0.010	0.054	0.010	0.056
	2,000	Q	0.25	0.006	0.036	0.005	0.037
			0.5	–0.012	0.039	–0.012	0.039
			0.75	–0.021	0.043	–0.022	0.044
		M		0.005	0.041	0.006	0.037

*Q, Q-MLIRT model; M, M-MLIRT model.*

**TABLE 4:** Bayesian estimates of regression coefficients when *K* = 40.

Case	n	Method	τ	β_1τ_		β_2τ_	
				Bias	RMSE	Bias	RMSE
1	500	Q	0.25	–0.021	0.075	–0.024	0.074
			0.5	–0.037	0.075	–0.037	0.078
			0.75	–0.038	0.079	–0.034	0.081
		M		0.012	0.078	0.013	0.077
	1,000	Q	0.25	–0.023	0.057	–0.023	0.056
			0.5	–0.025	0.060	–0.026	0.060
			0.75	–0.021	0.058	–0.019	0.055
		M		0.008	0.054	0.008	0.052
	2,000	Q	0.25	–0.011	0.040	–0.014	0.039
			0.5	–0.013	0.040	–0.015	0.041
			0.75	–0.012	0.040	–0.015	0.037
		M		0.006	0.039	0.008	0.040
2	500	Q	0.25	–0.026	0.075	–0.027	0.074
			0.5	–0.047	0.084	–0.040	0.083
			0.75	–0.036	0.073	–0.035	0.072
		M		–0.042	0.086	–0.037	0.088
	1,000	Q	0.25	–0.024	0.059	–0.027	0.060
			0.5	–0.020	0.057	–0.020	0.056
			0.75	–0.028	0.058	–0.029	0.058
		M		–0.047	0.069	–0.044	0.068
	2,000	Q	0.25	–0.024	0.045	–0.023	0.045
			0.5	–0.018	0.042	–0.019	0.043
			0.75	–0.025	0.046	–0.024	0.046
		M		–0.052	0.061	–0.051	0.060
3	500	Q	0.25	0.014	0.066	0.015	0.067
			0.5	–0.021	0.068	–0.020	0.066
			0.75	–0.024	0.073	–0.035	0.076
		M		0.015	0.075	0.014	0.075
	1,000	Q	0.25	0.013	0.052	0.016	0.051
			0.5	–0.017	0.053	–0.013	0.051
			0.75	–0.021	0.060	–0.012	0.057
		M		–0.009	0.052	–0.006	0.054
	2,000	Q	0.25	0.011	0.035	0.009	0.035
			0.5	–0.003	0.035	–0.005	0.034
			0.75	–0.008	0.039	–0.009	0.040
		M		0.003	0.038	0.001	0.038

*Q, Q-MLIRT model; M, M-MLIRT model.*

When comparing the estimation of regression coefficients of the M-MLIRT model and the Q-MLIRT model, we find that: first, M-MLIRT slightly outperforms Q-MLIRT in case 1. This is because that the error distribution in case 1 is normal, which meets the assumption of the LR model, so we expect the M-MLIRT model to outperform in that case; second, for case 2, the regression coefficients estimated by the M-MLIRT model are worse than those by the Q-MLIRT model to some extent. Specifically, the results are similar at τ = 0.5 when the sample size is small, but with increasing sample size, there are larger bias and RMSE for the estimates of the M-MLIRT model than those of the Q-MLIRT model. This is mainly because the error is heteroscedastic and the distribution of θ_*i*_ is heavy-tailed, and LR models are sensitive to heavy-tailed distributions and extreme outliers. For case 3, the results are close to each other, although the error term has a skewed distribution. It is perhaps that the degree of skewness of the distribution in case 3 is insufficient to influence the estimation of the regression coefficients in MLIRT.

## Empirical Example

The main research question in this section is how the explanatory variables of interest regarding individual characteristics and family background are related to students’ achievement in mathematics. It is quite possible that the relation between explanatory variables and students’ achievement may differ across different levels of students’ achievement, while LR only provides us the information about the conditional mean of students’ achievement and ignores the characteristics of the entire distribution of students’ achievement. Thus, the proposed Q-MLIRT model is expected to achieve a more appropriate analysis.

### Data Source

In this section, a subset of the PISA 2018 mathematics data was analyzed. There were 24 test forms/booklets that contained clusters of items from the mathematics domain. Each test form included four clusters, two clusters of items from the reading domain and one or two clusters of items from the mathematics domain. We chose a group of students from the United States who were administered test forms 1 to 6 for analysis. Each of these forms contained two mathematics clusters. Therefore, there were six clusters (i.e., M01-M06) in which contained 64 mathematical items. Because each student received one of the forms from his or her assigned group, and each form contained different items, so each student answered about 20 items and each item was answered about by 375 students. The sample size was n = 840 after eliminating students with missing data, and there were K = 64 dichotomous items. More information on the PISA 2018 data can be found at www.oecd.org/pisa/data/2018database/.

Three individual and family background variables, that have been used in most studies (e.g., Adams et al., 1997; Fox and Glas, 2001; Fox, 2005; Lu et al., 2018), were chosen as explanatory variables of interest. The student-grade variable (X_1_) equaled −1, 0, and 1, which represented grade 9, grade 10, grade 11, respectively. The corresponding number of students in each grade was 59, 630, and 150. Since only one student was in grade 12, so her was removed, hence the final sample size was n = 839. The student-gender variable (X_2_) equaled 0 (female) or 1 (male), the corresponding number of students was 421 and 418. The ESCS (economic, social, and cultural status of parents) variable (X_3_) was normally standardized. Information of these explanatory variables are summarized in Table 5.

**TABLE 5:** Summary of explanatory variables.

**Label**	Description	**Mean**	**SD**
Grade (X_1_)	−1 = grade 9 0 = grade 10 1 = grade 11	0.1085	0.2376
Gender (X_2_)	0 = female 1 = male	0.4982	0.2503
ESCS (X_3_)	Normally distributed	0.1314	1.0356

### Method

The PISA 2018 technical report stated that the item parameters were calibrated by the two-parameter logistic model (2PLM) or the generalized partial credit model (GPCM). As only dichotomously scored items are considered, we use the 2PNO model here, and we fix the first discrimination parameter to one and the first difficulty parameter to zero to identify the model. To assess the effects of the explanatory variables above on students with different ability levels, the QR model is given by:

$$Q_{\tau }\,\left(\theta_{i}\right)={\text{X}}_{i}^{T}\,\beta_{\tau },$$

where *i* = 1,…,839, β_τ_ = (β_0τ_,β_1τ_,β_2τ_,β_3τ_)*T* is a 4 × 1 vector of unknown QR coefficients under the τth quantile of the ability distribution, **X**_*i*_ = (1,*X*_1*i*_,*X*_2*i*_,*X*_3*i*_)*T* is a 4 × 1 vector that represents the explanatory variables of student *i*, the distribution of the error term is assumed as *AL*(0,ω,τ). The estimation was conducted for the quantiles of τ = 5%, 10%, 25%, 50%, 75%, 90%, and 95% to obtain a more thorough overview of the relationship between these explanatory variables and students’ mathematical achievement.

In the Bayesian analysis, the hyperparameters of the prior distributions were given as assigned in the simulation study. In the implementation of the Gibbs sampling algorithm, 10,000 iterations were done to estimate the parameters with an initial burn-in of 5,000 iterations. Convergence of the chains was checked by PSRF, and the PSRF values were less than 1.10 in the burn-in phase for all parameters under each quantile.

The M-MLIRT model was also used to fit the data, and compared with the Q-MLIRT model using deviance information criterion (DIC, Spiegelhalter et al., 2002). The model with smaller DIC was recommended as a better-fitting one. The DIC calculated using the joint likelihood conditioned on parameters at all levels. For the Q-MLIRT model, the joint likelihood is:

(18) $$f\left(\theta ,\text{Z}|\xi ,\beta_{\tau },\omega ,e\right)=f\left(\text{Z}|\theta ,\xi \right)f\left(\theta |\beta_{\tau },\omega ,\text{e}\right),$$

and the joint DIC for the τth quantile of the Q-MLIRT model is defined as:

(19) $$D\,I\,C_{\tau }=-2\,l\,o\,g\,\left\{f\,\left(\theta ,\text{Z}|\xi ,\beta_{\tau },\omega ,e\right)\right\}.$$

For the M-MLIRT model, the joint likelihood is:

(20) $$f\left(\theta ,\text{Z}|\xi ,\beta ,\alpha^{2}\right)=f\left(\text{Z}|\theta ,\xi \right)f\left(\theta |\beta ,\alpha^{2}\right),$$

and the joint DIC is defined as:

(21) $$D\,I\,C=-2\,l\,o\,g\,\left\{f\,\left(\theta ,\text{Z}|\xi ,\beta ,\alpha^{2}\right)\right\}.$$

Interested readers can refer to Celeux et al. (2006); Wang et al. (2013), Zhang et al. (2019) for more information of the joint DIC.

### Results

**FIGURE 1:**
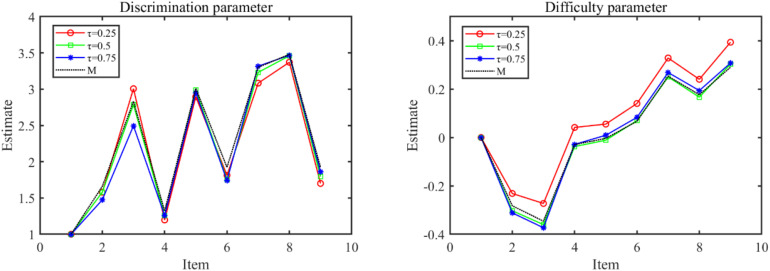
Item parameter estimates of the M-MLIRT(M) model and the Q-MLIRT model under different quantiles (τ = 25, 50, and 75%).

The parameter estimates of the Q-MLIRT model under various quantiles and the M-MLIRT model (reported in column “M”) are presented in Tables 6, 7. For the limited space, we only present the results of the item parameters of cluster 1 from form 1, there are 9 items. From Table 6, it is showed that the estimates of item parameters obtained based on the M-MLIRT model are close to the results obtained from the Q-MLIRT model, especially at 50% quantile. We calculated the cosine similarity between each two discrimination-parameter vectors and each two difficulty-parameter vectors estimated under different quantiles, and these values were all very close to 1, that means they are very similar to each other, and we can choose any group of them as the estimations. Figure 1 shows item parameter estimates of the M-MLIRT model and three selected groups of item parameters estimated under τ = 25%, 50%, and 75% of the Q-MLIRT model, which represent the corresponding results of the lower, middle, and higher quantiles, respectively. As can be seen, all the estimates of item parameters under different quantiles are close and of the same size order.

**TABLE 6:** Item parameter estimates of PISA data.

Estimation at different quantiles								
	M	5%	10%	25%	50%	75%	90%	95%
*a*_1_	1.000	1.000	1.000	1.000	1.000	1.000	1.000	1.000
*a*_2_	1.660	0.943	1.249	1.579	1.580	1.473	1.278	1.067
*a*_3_	2.831	1.828	2.412	3.006	2.786	2.495	2.083	1.727
*a*_4_	1.319	0.710	0.937	1.196	1.260	1.258	1.080	0.893
*a*_5_	3.011	1.743	2.323	2.896	2.989	2.951	2.527	2.107
*a*_6_	1.922	1.105	1.474	1.812	1.758	1.740	1.511	1.239
*a*_7_	3.302	1.851	2.484	3.083	3.232	3.317	3.038	2.688
*a*_8_	3.480	2.143	2.791	3.371	3.460	3.468	3.190	2.832
*a*_9_	1.923	1.008	1.359	1.700	1.795	1.863	1.701	1.452
*b*_1_	0.000	0.000	0.000	0.000	0.000	0.000	0.000	0.000
*b*_2_	–0.282	–0.253	–0.307	–0.231	–0.303	–0.312	–0.392	–0.439
*b*_3_	–0.346	–0.327	–0.361	–0.273	–0.360	–0.373	–0.470	–0.539
*b*_4_	–0.029	0.195	0.033	0.042	–0.037	–0.028	–0.066	–0.055
*b*_5_	–0.004	0.211	0.045	0.056	–0.011	0.009	–0.012	0.012
*b*_6_	0.067	0.354	0.151	0.141	0.070	0.083	0.063	0.103
*b*_7_	0.253	0.666	0.388	0.329	0.250	0.269	0.276	0.349
*b*_8_	0.177	0.504	0.271	0.241	0.167	0.194	0.196	0.256
*b*_9_	0.291	0.788	0.477	0.394	0.304	0.308	0.311	0.392

**TABLE 7:** Structural regression coefficient estimates of PISA data.

τ	β_0τ_	SD	β_1τ_	SD	β_2τ_	SD	β_3τ_	SD
5%	–0.474	0.053	0.237	0.041	0.033	0.031	0.170	0.024
10%	–0.432	0.042	0.185	0.028	0.033	0.025	0.131	0.018
25%	–0.235	0.052	0.177	0.024	0.049	0.023	0.115	0.013
50%	–0.133	0.058	0.190	0.025	0.077	0.021	0.117	0.013
75%	0.070	0.040	0.196	0.026	0.098	0.023	0.125	0.013
90%	0.210	0.046	0.213	0.029	0.109	0.026	0.144	0.015
95%	0.337	0.044	0.238	0.030	0.131	0.030	0.168	0.016
M	–0.120	0.054	0.181	0.025	0.068	0.023	0.121	0.013

The relationship between students’ mathematical achievement and the predictors can be deduced from the structural regression coefficients, the estimates of the QR and LR coefficients associate with the posterior standard deviations (SD) are showed in Table 7. The results of the M-MLIRT model show that, on average, the student grade and family background (i.e., ESCS) variables have significantly positive correlations with students’ mathematical achievement. Specifically, students in higher grades perform better, and students whose parents have higher economic, social, and cultural status have better mathematical achievement. But for gender, results of M-MLIRT show that there is a weakly positive correlation between gender and mathematical achievement, the performance of male students is slightly better than female students.

**FIGURE 2:**
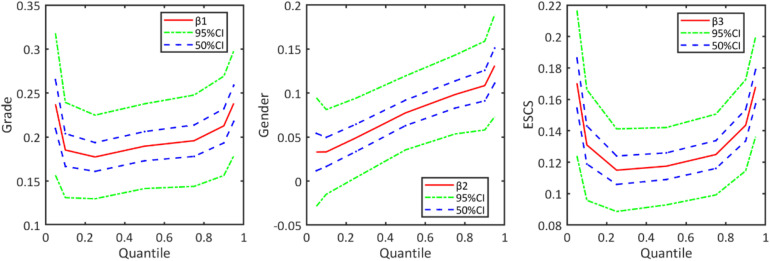
Regression coefficients of Q-MLIRT model across different quantiles in the analysis of PISA data, along with the corresponding 50 and 95% credible intervals.

When the results of M-MLIRT and Q-MLIRT are compared, it is argued that there are several differences between these two models. The results of the Q-MLIRT model show that relationships between these explanatory variables and students’ mathematical achievement are different across the achievement distribution. First, we find positive correlations between those explanatory variables and mathematical achievement at various locations along the distribution, and the results of Q-MLIRT at 50% quantile are as close as the M-MLIRT. For student grade and family background variables, it is found that the positive correlations between them and mathematical achievement are more significant at lower and higher quantiles of the distribution, that is the relationships between them and mathematical achievement are stronger in students with high-ability and low-ability. Regarding gender, we find that the QR structural coefficient of gender increases from 5% quantile towards the 95% quantile of the achievement distribution, which means that male students perform better than female students in mathematics, and the difference is exacerbated among high-ability students. This finding is consistent with González de San Román and De La Rica (2012). They analyzed gender gaps in PISA test scores in their study, and showed that in most PISA participating countries, male students outperformed than female students in mathematical achievement, moreover, gender gap in test scores differed significantly in different parts of the test score distribution, where the gap increased from the lower tail to the upper tail. The structural regression coefficients across the various quantiles are illustrated further in Figure 2.

In addition, Table 8 shows a comparison of the Q-MLIRT model and the M-MLIRT model based on the joint DIC. Results indicate that for the Q-MLIRT model, 25% quantile is the best-fitting, followed by quantiles at 75%, 50%, 10%, 5%, 90% and 95% ones. And the fitting situation of M-MLIRT is nearly equally to the Q-MLIRT model when the quantile is 50% or 75%, this is consistent with what is found in the estimation of parameters, that the parameter estimation results of M-MLIRT is mostly close to the results of Q-MLIRT at 50% and 75% quantiles.

**TABLE 8:** Comparing the Q-MLIRT and M-MLIRT using joint DIC for PISA data.

M-MLIRT	DIC of Q-MLIRT at different quantiles						
	5%	10%	25%	50%	75%	90%	95%
51926.1	52012.9	51990.1	51859.7	51980.2	51971.5	52076.0	52189.4

To sum up, the analysis results make it clear that the Q-MLIRT model offers a more comprehensive picture of the relationship between students’ mathematical achievement and the student-background variables. The results suggest that the relationships between students’ mathematical achievement and its predictors differ across different levels of student ability, which can’t be achieved with MLIRT. We find that for students with lower and higher mathematical performances in PISA, their achievements are related more closely to grade, and the socioeconomic status of their family, and the gender difference is more pronounced among high-level students. In this respect, Q-MLIRT is a useful research tool to better understand the relationship between students’ individual characteristics or family background factors and educational achievement. Note that the results, together with some educational knowledge, would provide a deeper insight for related educators on mathematical learning, and these findings will also help them to develop more-targeted policies for students of different ability levels.

## Discussion

In this article, a quantile-regression-type structural model was introduced to MLIRT models. The main advantage of this form is that it offers a richer analysis of the relationship between covariates and latent variables. Meanwhile, Q-MLIRT is more robust and is applicable to various distribution of latent variables. In other words, it is more flexible and applicable in general. We also proposed a fully Bayesian estimation algorithm for the Q-MLIRT model so that the parameters in the model could be estimated simultaneously. In addition, we proposed a new evaluation index based on the cosine similarity function to evaluate further the accuracy of the item parameter estimates under different quantiles.

In the simulation study, we used Gibbs sampling to estimate the Q-MLIRT model. The results showed that the parameters could be recovered well in almost all conditions. The Q-MLIRT model was applied to analyze a PISA data set, and the results provided more specific and in-depth insights into the relationship between the explanatory variables of interest and students’ achievement. Also, note that the item parameter estimates differ for different quantiles, although we know they are very close to each other according to the cosine similarity evaluation. An alternative and easy way of applying the Q-MLIRT model in practical problems is to obtain the item parameters at a certain quantile first, such as τ = 0.5 of the model, and then, for analysis with a different value of τ, the item parameters are pre-fixed (Wang et al., 2016).

This study was the first attempt at combining the QR approach with the IRT model in a multilevel modeling framework. However, this study focused only on item response data nested within students. We intend to extend the model to a higher level (school level) and estimate how the school level affects students’ abilities. Moreover, we considered herein only dichotomous response data and one-dimensional ability parameters, whereas Q-MLIRT could be applied to multidimensional and/or polytomous data. In addition, the explanatory variables that we have considered are known covariates, whereas it would make sense to consider latent explanatory variables measured by observations with measurement errors. Finally, variable selection would be of interest applied in Q-MLIRT, especially when some explanatory variables are either insignificant or significant only under some quantiles but not others.

## Data Availability Statement

Publicly available datasets were analyzed in this study. This data can be found here: https://www.oecd.org/pisa/data/2018database.

## Author Contributions

HZ did the simulation study and analysis on real data, and completed the writing of the article. WG provided key theoretical and technical support. XZ provided original thoughts and article revisions. All authors contributed to the article and approved the submitted version.

## Conflict of Interest

The authors declare that the research was conducted in the absence of any commercial or financial relationships that could be construed as a potential conflict of interest.

## Appendix

Here we give more details about the ALD and the Bayesian QR method. We consider a general QR model given by:

(A1) $$y_{i}={\text{d}}_{i}^{T}\,{\text{r}}_{\tau }+\varepsilon_{i},i=1,\ldots ,n,$$

where *y_i* and **d**_*i*_ denote response variable and a 4 × 1 vector of covariates for the *i*th observation, **r**_τ_ is a 4 × 1 vector of QR coefficients under the τth quantile of *y_i*, and the τth quantile of ε_*i*_ is assumed to be zero. The QR model does not have a specific likelihood function because the distribution of the error term ε_*i*_ is not specified. Yu and Moyeed (2001) first proposed Bayesian QR methods, they assigned each error term an asymmetric Laplace distribution (ALD), and considered MCMC methods for posterior inference.

About the main properties of the *A**L**D*(μ,ω,τ), parameters μ and τ in ALD satisfy: μ is the τth quantile of the distribution, and if *X*∼*A**L**D*(0,1,τ), then *G* = μ + ω*X*∼*A**L**D*(μ,ω,τ). In particular, the mean and variance of *g* are given by $E\,\left(g\right)=\mu +\frac{\omega \,\left(1-2\,\tau \right)}{\tau \,\left(1-\tau \right)}$ and $v\,a\,r\,\left(g\right)=\frac{\omega^{2}\,\left(1-2\,\tau +2\,\tau^{2}\right)}{{\left(1-\tau \right)}^{2}\,\tau^{2}}$ respectively. These important features of ALD have been generally adopted for the quantile inference (Yu et al., 2003). Further details about ALD can be found in Yu and Zhang (2005).

By assuming the error terms in Eq. (A1) distributed as *A**L**D*(0,ω,τ), then *y_i* follows $A\,L\,D\,\left({\text{d}}_{i}^{T}\,{\text{r}}_{\tau },\omega ,\tau \right)$, in this case the likelihood function for the model (i.e., Eq. A1) is:

$$L\,\left(r;y,\tau \right)=\frac{\tau^{n}\,{\left(1-\tau \right)}^{n}}{\omega^{n}}\,e\,x\,p\,\left\{-\frac{\sum_{i=1}^{n}\rho_{\tau }\,\left(y_{i}-{\text{d}}_{i}^{T}\,{\text{r}}_{\tau }\right)}{\omega }\right\}.$$

Yu and Moyeed (2001) noted that the maximum likelihood estimation of **r**_τ_ is equivalent to the solution of the frequentist approach to the estimation of coefficients, which is to solve the following optimization problem as pointed out by Koenker and Bassett (1978):

$$\min_{r}\sum_{i=1}^{n}\rho_{\tau }\,\left(y_{i}-{\text{d}}_{i}^{T}\,{\text{r}}_{\tau }\right).$$

As discussed in Yu and Moyeed (2001), the use of ALD for the error terms provides a natural way to deal with the Bayesian QR problem.

Since ALD is not a standard distribution so that it lacks a conjugate prior, direct use of the likelihood above is rather inconvenient for Bayesian inference. In this case, Reed and Yu (2009); Kozumi and Kobayashi (2011) used a mixture representation of ALD to develop the Gibbs sampling algorithm to estimate the parameters in QR. Specifically, if a random variable *y* follows *A**L**D*(μ,ω,τ), then it can be represented as the following form:

(A2) $$y=\mu +k_{1}\,e+\sqrt{k_{2}\,\omega \,e}\,\sigma ,$$

where $k_{1}=\frac{1-2\,\tau }{\tau \,\left(1-\tau \right)}$, $k_{2}=\frac{2}{\tau \,\left(1-\tau \right)}$, *e* follows exponential distribution with scale parameter ω, and σ is a standard normal distribution. That is to say, *y* is augmented by a latent variable *e*, thus the conditional distribution of *y* is normal with mean μ + *k*_1_*e* and variance *k*_2_ω*e*. It has been shown that the mixture representation provided fully conditional posterior densities and simplified the existing estimation procedures for QR models.

^1^A random variable *g* is said to follow *A**L**D*(μ,ω,τ) if it has density $f\,\left(g|\mu ,\omega ,\tau \right)=\frac{\tau \,\left(1-\tau \right)}{\omega }\,e\,x\,p\,\left\{-\rho_{\tau }\,\left(\frac{g-\mu }{\omega }\right)\right\}$, where μ is the location parameter, ω is the scale parameter, τ is the skewness parameter, and ρ_τ_(*x*) = *x*(τ−*I*(*x* < 0)) is called the check function.
